# Modulating neuroinflammation through molecular, cellular and biomaterial‐based approaches to treat spinal cord injury

**DOI:** 10.1002/btm2.10389

**Published:** 2022-08-31

**Authors:** Cheryl Yi‐Pin Lee, Wai Hon Chooi, Shi‐Yan Ng, Sing Yian Chew

**Affiliations:** ^1^ Institute of Molecular and Cell Biology A*STAR Research Entities Singapore Singapore; ^2^ School of Chemical and Biomedical Engineering Nanyang Technological University Singapore Singapore; ^3^ Lee Kong Chian School of Medicine Nanyang Technological University Singapore Singapore; ^4^ School of Materials Science and Engineering Nanyang Technological University Singapore Singapore

**Keywords:** central nervous system, inflammatory response, nerve regeneration, neural tissue engineering, tissue engineering scaffolds

## Abstract

The neuroinflammatory response that is elicited after spinal cord injury contributes to both tissue damage and reparative processes. The complex and dynamic cellular and molecular changes within the spinal cord microenvironment result in a functional imbalance of immune cells and their modulatory factors. To facilitate wound healing and repair, it is necessary to manipulate the immunological pathways during neuroinflammation to achieve successful therapeutic interventions. In this review, recent advancements and fresh perspectives on the consequences of neuroinflammation after SCI and modulation of the inflammatory responses through the use of molecular‐, cellular‐, and biomaterial‐based therapies to promote tissue regeneration and functional recovery will be discussed.

## 1 INTRODUCTION

Spinal cord injury (SCI) is one of the leading causes of long‐term physical impairment, with an increase in global prevalence of approximately 368 per 100,000 patients over the last 30 years.^1^ According to epidemiological studies, the most common causes of SCI across all populations result from falls, sports‐related injuries, and traffic accidents.^2^, ^3^, ^4^, ^5^ Patients with SCI usually suffer from temporary or permanent disabilities including loss of motor, sensory, and autonomic functions, and at times experience psychological stress, such as depression or a change in personality.^6^ While these effects are debilitating for the patients, the consequences of SCI also extend to the families in the form of caretaking assistance and financial dependency.

Self‐recovery of neural functions after complete SCI is rare, in part due to a lack of plasticity and limited regenerative capacity of the neural tissues.^7^ Following an initial mechanical injury that leads to tears, compression, and distortion to the spinal cord, vascular changes characterized by vasodilation, hyperemia, and petechial hemorrhages occur.^8^ Progressively, the spinal cord undergoes a series of cellular and molecular changes, including edema, gliosis hyperplasia, formation of an intrinsic inhibitory environment, scarring, and neuroinflammation, which would hinder axonal regeneration.^8^, ^9^, ^10^ Current SCI treatments remain palliative in the form of stabilization, surgical decompression, medication, and rehabilitation.^5^ The complex and dynamic pathophysiological events after SCI, especially a cascade of immunological responses resulting from neuroinflammation, pose as a major challenge for many therapeutic interventions, including cell‐ and biomaterial‐based therapies.^11^ Thus, a comprehensive review of the recent advancements in the cellular and molecular mechanisms involved in neuroinflammation after SCI is crucial to develop strategic interventions against this debilitating condition.

## 2 NEUROINFLAMMATION AFTER SCI

Neuroinflammation is defined as an inflammatory response that occurs within the brain or spinal cord. Upon damage to the blood‐spinal cord barrier (BSCB) after a physical trauma, neuroinflammation is one of the key components during the primary phase, which persists towards the secondary phase of injury.^8^, ^12^ The acute period of neuroinflammation is characterized by an infiltration of neutrophils and monocytes to the site of injury,^13^ whereas in the chronic phase, the progressive tissue degeneration that takes place across a period of months is primarily driven by lymphocytes.^14^ Inflammatory responses play a central role in regulating the pathophysiology after SCI, which greatly contributes to the repair of damaged tissues.^15^, ^16^ However, excessive inflammation may also lead to apoptosis of neurons and oligodendrocytes, resulting in a decline in neuronal functions.^16^ Inevitably, changes within the spinal cord microenvironment during neuroinflammation may aggravate and accelerate the course of SCI.

## 3 MICROENVIRONMENT CHANGES DURING NEUROINFLAMMATION

**FIGURE 1:**
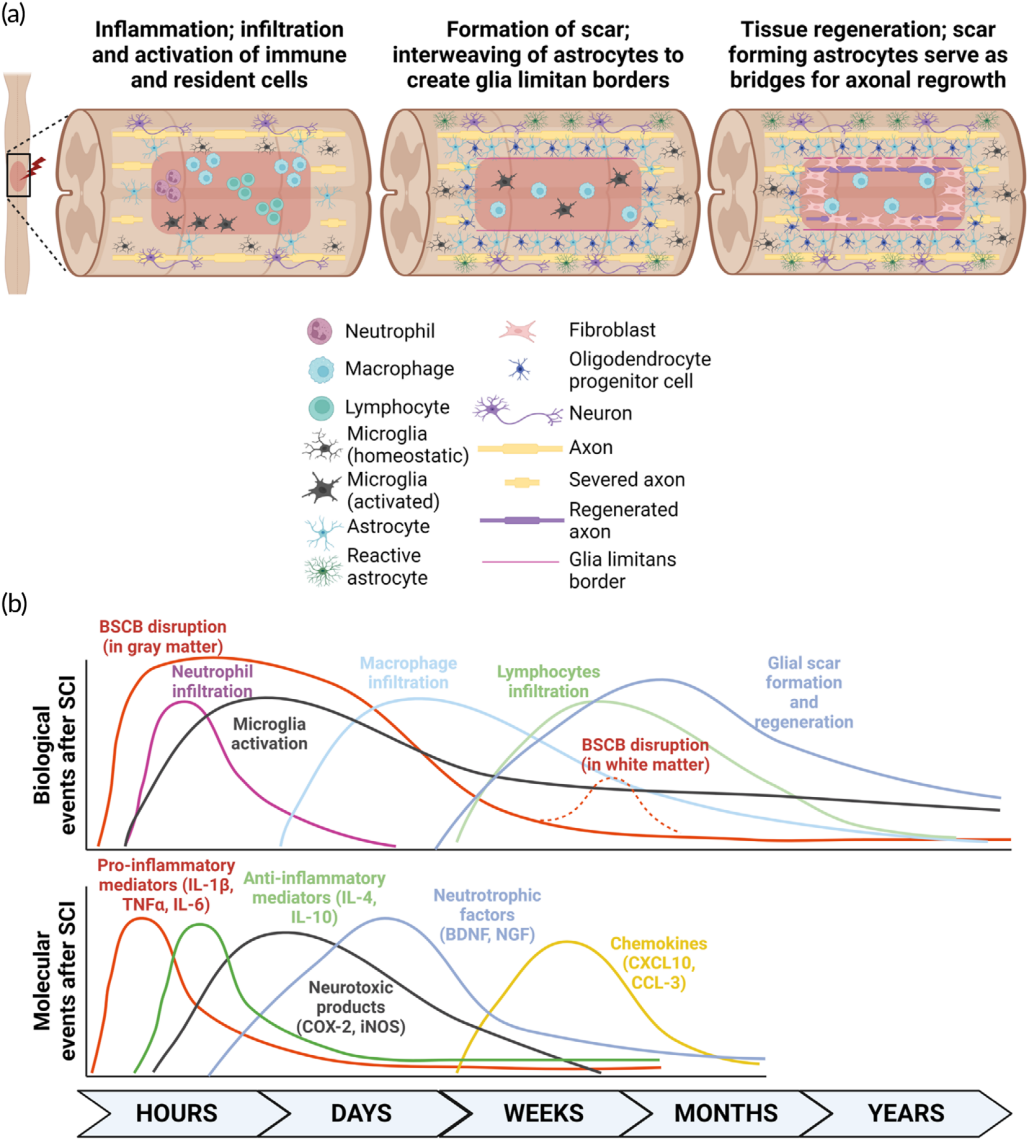
Schematic of the spinal cord microenvironment after spinal cord injury (SCI). (a) Within the first few hours after injury, inflammation occurs when peripheral immune cells begin infiltrating the lesion site, and resident immune cells become activated. Progressively, peri‐lesion perimeters with multicellular components including astrocytes, neurons, macrophages, microglia, oligodendrocyte progenitor cells, fibroblast, and activated astrocytes start to form a compact astrocyte core, regulating the formation of a glial scar to restrict inflammation and protect the surrounding of the injured tissue. These scar‐forming astrocytes serve as bridges for axonal regrowth, and structural tissue regeneration occurs weeks to months after SCI. (b) Timeline of both biological and molecular events following SCI. Illustrations are adapted from Donnelly and Popovich^41^ and created with BioRender.com.

During neuroinflammation, a cascade of cellular and molecular inflammatory pathways is activated, which includes the influx of circulating immune cells (neutrophils, monocytes, and lymphocytes), activation and proliferation of resident microglia and astrocytes, and the production of several mediators such as cytokines, chemokines, and reactive oxygen species by immune cells that reside in the central nervous system (CNS; Figure 1).^8^, ^12^, ^14^, ^17^ Paradoxically, while these secreted molecules are important in re‐establishing tissue homeostasis and assisting in wound healing and repair,^18^, ^19^ there are also collateral effects of secondary damage by inhibiting axonal regeneration or causing neuronal hypersensitivity, leading to neuropathic pain.^20^, ^21^, ^22^ Together, this imbalance may impair regenerative capacity and functional recovery.

### 3.1 Cellular imbalance

#### 3.1.1 Peripheral immune cells

Within a few hours after SCI, the first immune cell type to arrive at the site of injury is the neutrophils.^12^ They secrete oxidative and proteolytic enzymes to sterilize the lesion and prepare the tissue for subsequent repair.^12^ However, their presence is short‐lived approximately 3–5 days, plausibly due to their neurotoxic nature as neutrophils release potent free radicals.^8^, ^12^ A few days after neutrophils infiltration, monocyte‐derived macrophages are recruited to phagocytose dead cells including apoptotic neutrophils from the lesion.^23^ Interestingly, these macrophages have been reported to also secrete factors such as resolvins and protectins to prevent further recruitment of neutrophils to the damaged tissue.^24^ Unlike neutrophils, macrophages reside in the SCI lesions for as long as a year in humans.^12^, ^25^ While the recruitment of these innate immune cells serves to promote neuronal regeneration, wound healing, and tissue repair, both cell types instinctively produce proteases including matrix metalloproteinases (MMP), and oxidative metabolites that would compromise the BSCB.^12^, ^26^, ^27^

On the other hand, adaptive immune cells such as the T‐ and B‐lymphocytes also infiltrate the lesion site, albeit only after weeks to months later.^12^, ^14^ SCI‐induced T‐lymphocytes typically have a life span of 1–2 months and are also involved in the recovery and regeneration of the spinal cord tissues.^28^, ^29^ Reportedly, T‐lymphocytes elicit their neuroprotective capability through the recognition of specific neural antigens, such as myelin basic protein (MBP), whereby a drastic improvement in the rate of neuronal survival was observed.^30^, ^31^ However, even though T‐lymphocytes are relatively lower in numbers than macrophages, they are also capable in inflicting tissue damage, albeit controversially, through the recognition of the same MBP antigen.^32^ These opposing outcomes arise, depending on the spinal cord microenvironment at the time of injury, which would drive the equilibrium towards either a pathogenic Th1 or immunoregulatory Th2 lymphocytes expansion.^33^ For instance, in the event of more regulatory T‐lymphocytes recruitment to the lesion, there could be a more robust expression of neurotrophins, which would ameliorate the tissue damage induced by the secreted pro‐inflammatory cytokines.^34^ In association to an increase in T‐lymphocytes infiltration, there is an acute upregulation of cell death‐related genes and potassium voltage‐gated channel‐related (K_v_) genes.^35^, ^36^ The high expression of K_v_ genes such as contactin‐2 (*CNTN2*) typically occurs in response to early demyelination in rats.^36^ Furthermore, chronic T‐cell activation is shown to be involved in pathological tissue fibrosis and scarring.^37^

Since neural gene‐specific proteins such as anti‐MBP antibodies are detected after SCI, B‐lymphocytes are also involved during neuroinflammation.^32^ Mice deficient in B‐lymphocytes exhibited an improved locomotor function and reduced spinal pathology, indicating a pathogenic role of these cells in spinal cord tissue repair.^38^ The antibodies produced by SCI‐induced B‐lymphocytes are shown to be neurotoxic as the passive transfer of sera from SCI animals induced glial reactivity that is accompanied by prominent neuron loss.^14^ Interestingly, concomitant tissue injury may induce anti‐CNS antibodies that are able to promote axonal regeneration and remyelination.^14^, ^39^ For instance, antibodies targeting myelin may cause spinal cord demyelination, however, some antibodies prevent the binding by other myelin proteins that are inhibitory to axon growth and remyelination.^14^, ^40^ Together, there is a significant and long‐term contribution of peripheral immune cells during neuroinflammation within the spinal cord microenvironment.

#### 3.1.2 Resident immune cells of the CNS

Apart from the peripheral circulating innate and adaptive immune cells, resident cells of the CNS, such as microglia and astrocytes, also play crucial roles during neuroinflammation after SCI. Having the same progenitor as tissue macrophages, the microglia comprise 10% of the population in the CNS.^42^ These cells perform primary immunosurveillance functions of the tissue microenvironment, where they become elevated on the first day after SCI, and rapidly induce the production of cytokines and chemokines to recruit peripheral macrophages to the site of injury.^43^, ^44^, ^45^ Trophic factors secreted by microglia are necessary for the survival and proliferation of infiltrating cells, as well as the growth and regeneration of axons in the spinal cord lesion.^46^, ^47^ At the same time, microglia may also help to prevent further expansion of the lesion site.^48^ While the microglia responding to the damage after SCI is associated with tissue reorganization, it was reported to impede functional recovery of the neural tissue through the production of MMP‐9, which has been widely reported to amplify pro‐inflammatory cytokine secretion and affect the BSCB integrity, thereby interfering with plasticity and recovery.^49^, ^50^

Astrocytes are found in two areas of SCI lesion: (1) tissues that are spared by injury and (2) scar borders. The phenotype and functions of the astrocytes are distinct in both compartments.^51^ Astrocytes that reside in spared tissues are reactive, non‐proliferative, and hypertrophic, and they primarily intermingle with neurons and synapses.^51^ These hypertrophic astrocytes interact closely with neurons to promote axon sprouting and synapse plasticity through regulating the expression of neurocan, tenascin‐C, or directly producing thrombospondin‐1.^52^, ^53^, ^54^ On the other hand, scar‐forming astrocytes are majority spontaneously proliferated upon damage, where they interweave to create glia limitans borders that restrict inflammation and keep non‐neural lesion core apart from adjacent functioning spinal cord tissue.^55^, ^56^ Surprisingly, axonal regeneration is not impeded by the presence of astrocyte scar formation as these scar‐forming astrocytes may serve as bridges for axonal growth.^57^ Instead, the disruption of the scar tissues, shown through the use of loss‐of‐function transgenic mice that selectively kill proliferating scar‐forming astrocytes, led to an attenuation of axon growth after SCI.^58^

Astrocyte scar borders are intertwined with reactive oligodendrocyte progenitor cells that express neuron glial antigen 2 (NG2‐OPCs). Similarly, NG2‐OPCs are also present in both the spared tissues and scar borders. However, there have been several conflicting studies on axonal regrowth by these hypertrophic NG2‐OPCs within the scar borders,^59^, ^60^, ^61^, ^62^, ^63^ which warrant further investigations to understand the roles of these cells during neuroinflammation. Overall, the roles of these spinal cord neural cells play an important role in regulating tissue damage after SCI.

### 3.2 Molecular imbalance

#### 3.2.1 Cytokines and chemokines

Cytokines are regulatory mediators that contribute immensely during neuroinflammation, neurodegeneration, and neuropathic pain through intricate cross‐talks and interplays.^64^ They are usually classified into pro‐inflammatory or anti‐inflammatory proteins,^64^ although some cytokines may exhibit pro‐inflammatory and anti‐inflammatory properties under various circumstances.^65^ Endogenous cells in the spinal cord, mainly the neurons, microglia, and astrocytes, support the early production of key inflammatory mediators, such as interleukin (IL)‐1β, IL‐6, and tumor necrosis factor‐alpha (TNFα).^66^, ^67^, ^68^, ^69^ These pro‐inflammatory cytokines, along with others including granulocyte‐macrophage colony‐stimulating factor (GM‐CSF) and leukocyte inhibitory factor (LIF), contribute to the dynamic imbalance within the spinal cord microenvironment.^66^, ^67^, ^70^ At low concentrations, these cytokines elicit protective functions by inducing neurotrophic factors and adhesion molecules on the cell surface, which assist in leukocyte recruitment to the injury site.^71^ However, at a higher concentration, their pro‐inflammatory nature typically causes neuronal damage and destruction through the activation of transcription factors that stimulate the expression of neurotoxic genes such as cyclooxygenase 2 (COX‐2) and inducible nitric oxide synthase (iNOS).^72^, ^73^ High amounts of IL‐1 within the spinal cord microenvironment result in increased vascular permeability and lymphocyte recruitment, while IL‐6 promotes the activation and infiltration of peripheral immune cells and microglia.^74^ Blockade of IL‐6 signaling was reported to enhance SCI recovery as it abrogates damaging inflammatory activity and reduces the severity of connective tissue scar formation.^74^, ^75^ TNFα is involved in several aspects of SCI neuroinflammation. Upon secretion, TNFα promotes the extravasation of neutrophils to the damaged tissue through adhesion molecules including vascular cell adhesion molecule‐1 (VCAM‐1) and intracellular adhesion molecule‐1 (ICAM‐1).^76^ TNFα also induces changes to the permeability of endothelial cells, thereby compromising the integrity of the BSCB.^76^ In addition, this pro‐inflammatory cytokine exerts cytotoxic effects on oligodendrocytes, resulting in demyelination.^76^ Furthermore, TNFα also contributes to fibrotic scarring by stimulating the proliferation and hypertrophy of astrocytes.^77^

Anti‐inflammatory cytokines including IL‐4 and IL‐10 are also produced to regulate and aid in functional recovery after SCI.^78^ IL‐4 is secreted by activated T‐lymphocytes and is involved in the Th2 immunoregulatory pathway where it regulates the activation of acute macrophages and restrict secondary cavity formation after SCI.^79^ In addition, IL‐4 also drives microglia and macrophages toward an anti‐inflammatory phenotype that reduces tissue damage, thereby leading to an improved functional recovery.^80^ The production of IL‐10 by monocytes/macrophages, astrocytes, and microglia functions to suppress the inflammatory responses through the reduction of TNFα, IL‐1β, S100β, and iNOS.^15^, ^78^, ^81^ IL‐10 is involved in regulating the influx and efflux of macrophages out of the injured nerve, reducing the production of pro‐inflammatory chemokines and cytokines, and it is necessary for myelin‐phagocytosis‐induced shift of macrophages from pro‐inflammatory to anti‐inflammatory.^82^ Furthermore, the loss of IL‐10 affects axon regeneration, resulting in a poor recovery of motor and sensory functions.^82^ More recently, a scaffold that comprise photocrosslinked gelatin hydrogel that was incorporated with polyamidoamine and IL‐10 enhanced tissue remodeling and promoted axonal regeneration.^83^

On the other hand, chemokines are small, secreted molecules that stimulate specific functions during inflammation. The kinetics of chemokine production usually parallel the infiltration of immune cells after SCI.^45^ Chemokines that belong to the α family (CXC) primarily participate in chemotaxis functions, whereas those in the β family (CC) provide priming signal for immune cells.^76^ For instance, CXCL10 is involved in T‐lymphocyte recruitment after SCI, which contributes to post‐traumatic tissue loss,^84^ while CCL3 enhances the production of other pro‐inflammatory cytokines through the G‐protein coupled receptors CCR1, CCR4, and CCR5, leading to an exacerbation of inflammation that contributes to secondary tissue damage after SCI.^85^ Taken together, the unregulated production of inflammatory mediators, albeit molecularly small, can lead to disastrous consequences toward functional recovery after SCI.

#### 3.2.2 Neurotrophic factors

The levels of growth promoting and inhibiting factors become disproportionate after SCI, resulting in an inhibitory environment within the spinal cord tissue. Neurotrophic molecules have been reported to enhance the survivability and proliferation capacity of neural cells and axonal regeneration within the spinal cord.^86^ As such, an imbalance in these factors can lead to oligodendrocyte and neuronal death, as well as axonal degeneration. The most common neurotrophic factors include brain‐derived neurotrophic factor (BDNF), nerve growth factor (NGF), and neurotrophin‐3 (NT‐3).^86^, ^87^ These neurotrophic mediators are synthesized as pro‐peptides, which are cleaved intracellularly into mature neurotrophic proteins.^88^

BDNF is a key molecule that plays a neuroprotective role by regulating synaptic plasticity and contributing to synaptic transmission.^89^ However, its expression level is reduced drastically after SCI, and the overexpression of BDNF alleviates neuroinflammation through the induction of tyrosine kinase receptor B and phosphorylated p38.^90^ NGF expression after SCI demonstrated improved behavioral outcomes by promoting axonal sprouting of the sensory afferents.^91^ However, NGF has also been associated with neuropathic pain after nerve injury, where the binding of NGF to its receptors activates several downstream signaling pathways including the MAPK pathway.^92^ This in turn led to the activation of NF‐kB p65, which promotes the production of pro‐inflammatory cytokines such as TNF‐α and IL‐1β, resulting in the development and maintenance of pain.^93^, ^94^ Interestingly, the pro‐peptide of NGF, which is secreted in abundance after traumatic injuries, has been shown to reduce the number of oligodendrocytes through p75.^87^, ^95^ In addition, the complex formed between the precursor of NGF with Sortilin and p75 also triggers an apoptotic cascade.^96^ Hence, the imbalance between neurotrophic factors and their precursors may also affect neural cell survival and death.

#### 3.2.3 Ionic imbalance

It is understood that biochemical events associated with secondary tissue damage include the disruptions of ionic homeostasis of K^+^, Na^+^, and Ca^2+^ ion channels.^97^ Following SCI, these channels are dysregulated due to damage to the cell membrane, as well as the release of pro‐inflammatory mediators by immune cells.^98^ Disrupting the myelin sheath of axons within the spinal cord tissue causes the imbalance of K^+^ channels, which leads to further demyelination.^99^ At the same time, the concentration of Na^+^ becomes upregulated intracellularly, while K^+^ and Mg^2+^ become upregulated extracellularly, which eventually results in cellular edema.^100^ This ionic imbalance further triggers intracellular phospholipase activity and acidosis.^101^ Specifically, damaged neurons after SCI release high concentrations of glutamate neurotransmitter, causing Ca^2+^ dysregulation, which compromises cellular machinery while increasing neural cell death.^102^, ^103^, ^104^ Overall, ion imbalance plays a vital role in regulating the pathophysiology changes after SCI.

## 4 MANIPULATING NEUROINFLAMMATION TO TREAT SCI

Extensive attempts have been made in modulating neuroinflammation to improve recovery after SCI, either through blockade of detrimental immune cell functions and neurotoxic pathways or enhancing the production of reparative and restorative cells and molecules. These approaches range from molecular‐, cell‐ or biomaterial‐based therapies that target different aspects of neuroinflammation after SCI.

### 4.1 Molecular‐ and cell‐based therapies to improve SCI recovery

#### 4.1.1 Depletion of immune cells and mediators

Therapeutic interventions that target specific cell types or intracellular signaling pathways have demonstrated positive prognosis in treating SCI. Neuroprotection can be achieved through the attenuation of peripheral immune cells infiltration by targeting adhesion molecules that are expressed on the surface of monocytes and/or neutrophils, which can rescue the capacity of donor cell populations to promote locomotor improvement after SCI.^105^, ^106^, ^107^, ^108^ For instance, antibodies that target CD11d/CD18 or α4β1 integrins expressed on monocyte, macrophages, and CD11d expressing microglia disrupt monocyte‐endothelial cell interactions and reduce both microglia and macrophage accumulation within the lesion site, leading to a reduction in tissue loss and increased functional recovery after SCI in rodent models.^109^, ^110^, ^111^, ^112^ The use of anti‐Ly6G antibodies to deplete neutrophils has also led to improved recovery outcomes.^108^ Depletion of both neutrophils and monocytes showed an early reduction in oxidative stress, nonheme iron, and expression of MMP‐9 and stabilization of the BSCB, and thus greatly promoting neurological healing.^107^ However, due to the double‐edged nature of neuroinflammation, some studies have also shown a negative impact on wound healing and neurological outcomes when neutrophils are depleted.^113^, ^114^

Depletion of B‐lymphocyte with therapeutic CD20 antibodies, such as rituximab or obinutuzumab, has also been used in modulating neuroinflammation and immunological events associated with SCI by reducing cell death and nitric oxide level.^115^ These monoclonal antibodies also inhibit constitutive NF‐kB signaling pathways by reducing the phosphorylation of components involved in the NF‐kB pathway.^116^ This is crucial as NF‐kB is one of the pivotal mediators of pro‐inflammatory gene expression, as well as the transcription of pro‐inflammatory cytokines, chemokines, and adhesion molecules.^117^ In addition, therapeutic CD20 antibodies also led to lower expressions of TNFα and IL‐1β, which are associated with damage after SCI.^76^, ^77^, ^115^ As B‐lymphocytes have a role in trafficking T‐cells into the CNS,^118^ earlier findings have indicated that treatment with CD20 antibodies also affected T‐lymphocyte activation, plausibly due to a decrease in antigen presentation by B‐lymphocytes after depletion.^119^ Meanwhile, directly depleting T‐lymphocytes by split‐dose gamma radiation after thymectomy in 4‐week‐old rats may also enhance neuronal survival after SCI.^120^

Other than depleting immune cells or their adhesion factors, inhibition of cytokines or chemokines is another approach for limiting leukocyte infiltration and alleviating neuroinflammation. For instance, treatment with a broad‐spectrum chemokine receptor antagonist, vMIP‐II, reduces leukocyte influx and astrogliosis, while increasing axon and myelin sparing, and neuronal survival.^121^, ^122^ In addition, blocking the pro‐inflammatory cytokine, IL‐6, that promotes macrophages activation may also improve SCI recovery. Specifically, the monoclonal antibody, MR16‐1, that targets IL‐6 cytokine leads to the reduction of iNOS‐ and CD16/32+ macrophages, while promoting arginase‐1‐ and CD206+ macrophages.^74^ Interestingly, the effects of IL‐6 inhibition are not only limited to macrophage or microglia, as it also alters astrocyte activation and ameliorates functional recovery after SCI.^75^, ^123^ Antagonizing CXCL10, the chemokine that is responsible for T‐lymphocyte recruitment, has led to reduced neuronal death, an increase in axonal regeneration, and improve functional recovery after SCI.^121^ Furthermore, anti‐CXCL10 treatment also decreases the number of macrophages and B‐lymphocytes.^124^ The use of infliximab, which targets the pro‐inflammatory cytokine, TNFα, as well as the genetic deletion of TNFα receptors drastically reduce neuroinflammation and oxidative injury while ameliorating neuropathic pain after SCI.^125^, ^126^ Exogenous administration of IL‐1 receptor antagonist also led to a reduction in apoptosis and blocks p38 mitogen‐activated protein kinase pathway.^127^ Collectively, these findings suggest that targeting the inflammatory pathways is an alternative to improve neuroprotection and recovery after SCI.

#### 4.1.2 Promoting or transplanting cells with reparative and restorative functions

Another approach to improve functions after SCI focuses on immunomodulation and promotion of reparative immune cells such as the anti‐inflammatory macrophages, either by pharmacological or transplantation therapies.

Pharmacological agents have been widely used to promote SCI recovery by reducing inflammation and redirecting immune cells toward the reparative pathway. One commonly used macrolide antibiotic, Azithromycin, has been reported to promote anti‐inflammatory macrophage activation, which limits the secondary injury process after SCI, leading to improved tissue recovery.^128^, ^129^ Another anti‐inflammatory drug, minocycline, when administered acutely in a SCI rodent model has efficiently modulated the resident microglia to reduce its pro‐inflammatory response while maintaining a pro‐regenerative environment.^130^ Exogeneous administration of Maresin 1, a highly conserved specialized pro‐resolving mediator, has been demonstrated to resolve inflammatory responses by downregulating pro‐inflammatory cytokines such as CXCL1, CXCL2, CCL3, CCL4, IL‐6, and CSF3, silencing major inflammatory intracellular signaling pathways such as STAT1, STAT3, STAT5, p38, and ERK1/2, as well as altering macrophage activation toward the anti‐inflammatory phenotype.^131^ A more recent and comprehensive review on other immunomodulatory agents in spinal cord injury can be found in Wu et al.^132^

Stem cell therapies have recently garnered attention for SCI treatment due to their capability to differentiate and replace degenerated neural cells.^133^ Transplanted stem cells have been shown to promote neuro‐ and vascular‐protective outcomes at different phases of SCI.^134^ In addition to reorganizing the neuronal network, these cells also reduce local and systemic inflammation, support axonal regeneration and synaptic sprouting, and reduce glial scars.^134^ The mechanisms of stem cell therapy are categorized into three distinct roles: (1) cell replacement, where transplanted cells differentiate into neuronal or vascular cells to compensate for the lost functions^135^, ^136^; (2) functional multipotency, where the secretion of trophic factors from transplanted cells contribute to new neuronal circuit regeneration^137^ and (3) stem cell regeneration, where the transplanted stem cells activate regeneration of host neuronal stem cells.^138^ Many stem cell types including mesenchymal stem cells, neuronal stem cells, olfactory ensheathing cells, and Schwann cells, have been extensively shown as promising cell sources for transplantation due to their capacity to ameliorate tissue damage and assist in functional recovery through immunomodulation, pro‐angiogenic signaling and neural differentiation.^134^, ^139^, ^140^ In addition, these stem cells secrete mediators and cell adhesion molecules that play fundamental roles in improving tissue repair and regeneration, involving the activation of endogenous anti‐inflammatory macrophages and microglia.^133^, ^141^, ^142^, ^143^ However, the inflammatory microenvironment of the injured spinal cord can limit the regenerative capacity of endogenous or transplanted cells and lead to allograft rejection.^144^, ^145^ Hence, the exogenous administration of drugs to diminish the detrimental functions of immune cells have greatly facilitated the efficacy of cell‐based therapies against SCI.^146^

One example is methylprednisolone (MP), which is widely known as a potent corticosteroid. MP has significant neuroprotective and immunosuppressive functions by triggering immune cells apoptosis and reducing inflammatory events.^147^, ^148^, ^149^ Furthermore, it was documented that MP can inhibit the lipid peroxidation process and protect oligodendrocytes from apoptotic‐mediated neuronal death after SCI.^150^ More importantly, through clinical trials and meta‐analysis, the use of MP has significantly improved motor scores in SCI patients.^151^, ^152^ However, according to the American Association of Neurological Surgeons/Congress of Neurological Surgeons (AANS/CNS), MP is only recommended as an option for acute spinal injury treatment, and should only be taken with the prior knowledge that the evidence suggesting harmful side effects is more consistent than any suggestion of clinical benefit.^153^

Cyclosporine A (CsA), a calcineurin inhibitor, is a potent inhibitor of T‐lymphocyte activation that is commonly used to prevent allograft rejection and graft‐versus‐host disease.^154^ However, contrasting findings on the effectiveness of CsA on the survival of grafted stem cells and improve functional recovery have arisen.^155^, ^156^ The difference in outcomes could be attributed to the source of stem cells and the type of animal models. Another calcineurin inhibitor, tacrolimus (FK506), also exhibits potent immunosuppressive properties that reduce the extent of secondary injury after SCI.^157^ Similar to CsA, FK506 also targets the T‐lymphocytes and inhibits their proliferation. A handful of studies on transplantation to treat SCI using various stem cells have reported the safety and efficacy of FK506 and its potency in promoting graft survival and improving motor functions.^158^, ^159^, ^160^ The benefits of these calcineurin inhibitors are further enhanced when used in combination.^161^, ^162^

However, there remain several challenges in drug delivery to ameliorate neuroinflammation. For instance, the majority of the noninvasive route of drug delivery is less efficient in accessing the CNS, including the spinal cord, due to the presence of a BSCB.^163^ In addition, most of the bioactive compounds that can pass through the BSCB are lipophilic, which may have reduced stability and half‐lives under physiological conditions, resulting in difficulties to maintain an optimal dosage.^164^ More importantly, drug diffusion within the host may lead to off‐target effects, which has been reported with corticosteroids, where patients experienced severe side effects such as seizure, pneumonia, and haematemesis.^165^ As clinical trials of corticosteroids in SCI have been relatively small, with an emphasis on subgroup effects, the use of corticosteroids in SCI should remain an area of controversy.^165^ Thus, the involvement of biomaterial‐based approaches may help overcome some of the challenges faced during drug delivery.

### 4.2 Biomaterial‐based therapies to modulate neuroinflammation and treat SCI

#### 4.2.1 Localized drug delivery

To tackle the challenges in drug delivery to the injured spinal cord, noninvasive strategies utilizing drug‐loaded nanoparticles have been developed to overcome the BSCB.^166^, ^167^, ^168^ In recent years, nanoparticles with neuroinflammation‐targeting designs allowed more targeted delivery and had led to better recovery.^169^, ^170^

On the other hand, although it is more invasive, delivering the drugs in situ can bypass the BSCB and reach the injured site directly. Combined with a controlled‐release mechanism, localized drug delivery can reduce the potential side effects of the immunomodulation drugs. For instance, loading anti‐inflammation drugs in scaffolds or combining drug‐loaded micro/nanoparticles with a hydrogel had demonstrated a reduction in microglia/macrophages activation and pro‐inflammatory interleukins by ensuring that the local concentration of the drug is high enough to have a therapeutic effect (Table 2).^171^, ^172^, ^175^, ^176^, ^177^, ^178^, ^180^, ^187^, ^189^, ^194^, ^196^ More importantly, the particles can be designed to selectively target the microglia/macrophages and control uptake kinetics by changing surface charge.^176^, ^197^ Other than low molecular weight anti‐inflammatory drugs, scaffolds loaded with growth factors, microRNAs, and anti‐inflammatory cytokine‐encoding lentivirus also showed promising effects in reducing macrophage/microglial activation and improving functional recovery.^185^, ^190^, ^191^ These growth factors and microRNAs also have a direct effect on stimulating nerve regeneration, which makes them ideal candidates that could have a synergistic effect in both anti‐inflammation and nerve regeneration.

#### 4.2.2 Scaffolds for cell delivery

In addition to drug delivery, tissue engineering scaffolds have emerged as a powerful platform in combination with cell‐based therapies as a form of regenerative intervention. A central component of tissue engineering is the use of biomaterials as a vehicle for cell transplantation by providing mechanical stability and support for cell adhesion and migration or recruiting endogenous progenitor cells from the surrounding tissues.^198^ When the scaffolds are used to deliver cells, biomaterial scaffolds and cells synergistically controlled immune response and tissue regeneration (Table 3).^199^, ^203^, ^204^, ^205^, ^207^, ^208^, ^209^, ^210^, ^211^, ^212^, ^213^ Notably, mesenchymal stem cells secrete immunomodulating substances such as exosomes and CCL‐2 to convert the macrophages/microglia into anti‐inflammatory phenotypes.^193^, ^200^, ^212^ However, some implanted materials can evoke the host inflammatory response as they are regarded as foreign bodies that have been introduced to the site of lesion.^214^ Hence, it would be highly beneficial to design the SCI scaffolds to be immunomodulatory through manipulating material chemistry and mechanical properties before combining with cells and drugs to achieve better recovery outcomes.

#### 4.2.3 Material chemistry

Traditionally, implantable biomaterials have been designed to be biocompatible by evading the immune system and minimizing foreign body responses. Earlier studies on implants in the CNS found that many of the materials and coatings might be pro‐inflammatory and have low biocompatibility.^215^ To improve material biocompatibility, low protein‐binding coatings such as alginate could be useful in reducing microglial attachment.^215^ However, such an approach also limits the attachment of other neural cells that are essential for regeneration. Consequently, the focus has shifted toward exploiting the properties of the biomaterials to modulate the immune response and immune cell phenotypes to achieve the desired outcomes such as better regeneration.^216^

While anti‐inflammatory effects were evaluated in most scaffolds in the form of reduced macrophage/microglial activation, more recent materials and scaffolds designed for SCI were increasingly assessing pro‐ and anti‐inflammatory phenotypic switching as a feature of immunomodulation. Thus far, the majority of the natural materials used including decellularized extracellular matrices (ECM), collagen, laminin, chitosan, hyaluronic acid (HA), gelatin, and fibrin have well‐documented biocompatibility and anti‐inflammatory effects (Table 1).^230^, ^231^, ^232^ Furthermore, some of these materials such as collagen, chitosan fragments, high molecular weight HA can reduce activation of macrophages, microglia, and astrocytes while polarizing macrophages toward the anti‐inflammatory phenotypes.^209^, ^217^, ^221^, ^233^, ^234^ Likewise, scaffolds developed based on decellularized tissue are rich with ECM proteins and hence can promote anti‐inflammatory macrophage polarization and recruit CD4+ Th2 T‐lymphocytes to provide a pro‐regenerative environment.^208^, ^219^, ^235^, ^236^, ^237^ This is particularly crucial for cell delivery where small molecules produced by activated T‐lymphocytes might be cytotoxic to the grafted cells.^238^

**TABLE 1:** Selected scaffold‐based approaches with immunomodulation features after spinal cord injury

Scaffold material	Drug	Cell	Model	Results based on immune response	References
*Natural materials*					
Fragmented chitosan hydrogel suspension (Chitosan–FPHS)	–	–	In vitro mouse monocytes, Rat T8–T9 hemisection	Polarized macrophage polarization towards anti‐inflammatory phenotypes with decreasing degree of acetylation (DA) and increasing chitosan concentration; Decreased M1 macrophages with low DA chitosan‐FPHS implant in vivo	217
Chitosan–FPHS	–	–	Rat T8–T9 hemisection	Increased levels of M2 marker protein CD206 and Arg1	218
Porcine brain‐derived decellularized extracellular matrix	–	–	In vitro rat macrophages, Rat T9 contusion	Increased Arg1+ M2 macrophages and IL‐10 expression; Decreased CD86+ macrophages and increased Arg1+ M2 macrophages in vivo	219
Injectable optimized acellular nerve graft	–	–	Rat C4–C5 contusion	Increased the number of CD206+ M2 macrophages and expressions of CD206, arginase‐1 and IL‐10	220
Methacrylated high molecular weight HA	–	–	Rat T7–T8 hemisection	Decreased ED1+ macrophages; Limited astrocyte activation and CSPG deposition	221
Acetylated dextran microspheres	–	–	Rat T10 contusion	Reduced GFAP+ astrocytes and CD68+ microglia; Reduced neuron death by sequestering glutamate and calcium ions	222
*Synthetic/hybrid materials*					
Imidazole‐polyorganophosphazenes (I‐5) hydrogel	–	–	Rat T10 contusion	Decreased Iba1+ cells; Hydrogel interacted with macrophages and activated macrophage‐mediated wound healing responses	223
Hyaluronan/poly(ethylene glycol) diacrylate (HA/PEGDA)	–	–	Rat T9–T10 hemisection	Decreased Iba1+ cells and reactive astrocytes	224
Hyaluronan/methyl cellulose (HA/MC)	–	–	Rat T7 post‐traumatic syringomyelia (compression followed by subarachnoid injection of kaolin)	Decreased CSPG deposition and IL‐1α cytokine level but did not decrease neutrophil or macrophage/microglial activation	225
Graphene oxide	–	–	Rat C6 hemisection	Decreased ED1+ and Iba1+ cells with the presence of M2 macrophages	226
Poly(hydroxybutyrate‐co‐hydroxyvalerate)/polylactic acid/collagen (PHBV/PLA/Col) membrane duraplasty	–	–	Rat T10 contusion	Decreased the expression of NLRP3, ASC, cleaved‐caspase‐1, IL‐1β, TNF‐α, and CD86 but increased the expression of CD206; Reduced the infiltration of CD86+ macrophages to the lesion site	227
PCL‐HA nanofiber‐hydrogel composite	–	–	Rat T9 contusion	Polarized Infiltrated macrophages towards M2 phenotype; M2 macrophages congregated in nanofiber‐rich areas	228
Aligned PEG tubes in fibrin	–	–	Mouse T9–T10 hemisection	No difference in number of CD45+ leukocytes, Arg1+ M2 macrophages, Lyg6+ neutrophils, CD4+ T cells; Increased CD11c+dendritic cells, F4/80+ macrophages	229

*Note*: The phenotypes of macrophages and microglia are presented as reported by the respective studies. In these studies, M1 typically refers to the pro‐inflammatory phenotypes whereas M2 typically refers to the anti‐inflammatory phenotypes.

Synthetic materials such as polyurethane (PU), polylactic acid (PLA), polylactic‐co‐glycolic acid (PLGA), polycaprolactone (PCL), graphene oxide, and imidazole‐polyorganophosphazenes, which have been used as scaffold materials for SCI regeneration, have also been assessed to reduce inflammation.^191^, ^194^, ^223^, ^226^, ^239^ Although the anti‐inflammatory macrophages were observed in some of these scaffolds, the mechanism of how the materials polarize the macrophages is less clear.^223^, ^226^ Long‐term evaluation is also needed to confirm that the products from polymers degradation do not elicit an additional inflammatory response. Furthermore, caution should be exercised regarding the hydrophilicity of the polymer surfaces as monocytes/macrophages adhere better onto hydrophobic surfaces.^240^, ^241^ Therefore, it is desired to use coatings or additives to better control the immune response towards the polymer surfaces. In particular, ECM proteins or ECM‐derived peptides, which are effective in modulating macrophages, T lymphocytes, and B lymphocytes towards the anti‐inflammatory phenotypes, could be used to modify polymer surfaces.^232^ Similarly, L1 cell adhesion molecules, which are natively found on cell surfaces, could reduce inflammatory microglial encapsulation in vivo when it was utilized as a coating.^242^

#### 4.2.4 Stiffness

Similar to the material chemistry of the SCI scaffolds, evaluations of the effect of scaffold mechanical properties on peripheral immune cell responses have been mainly performed on macrophages but are limited to other peripheral immune cells, such as neutrophils and lymphocytes. Nevertheless, the relationship between these immune cells and the mechanosensing of substrate stiffness is well‐established (Table 2), which could be referenced for SCI scaffold designs.^237^ Depending on the range of substrate modulus tested, stiffer substrates generally stimulate higher activation and secretion of pro‐inflammatory cytokines from macrophages (130–840 kPa), dendritic cells (2–50 kPa), and neutrophils (0.2–128 kPa from two studies).^243^, ^244^, ^245^, ^246^ On the other hand, substrate stiffness had contrasting effects on different characteristics of T‐ and B‐lymphocytes. For example, human CD4+ and CD8+ T‐lymphocytes were activated and produced more cytokines on a substrate with stiffness at around 100 kPa as compared to substrates with stiffnesses of 0.5 kPa, 6.4 kPa, or 2 MPa.^247^, ^248^ For B‐lymphocytes, antigens on the stiffer substrates stimulated stronger activation responses in the range of substrate modulus tested (2.6–1100 kPa from two studies). However, the stiffer substrate (1100 kPa) had weaker B‐lymphocyte proliferation responses and in vivo antibody responses as compared to the softer substrate (20 kPa).^249^, ^250^

**TABLE 2:** Selected drug‐loaded scaffold‐based approaches with immunomodulation features after spinal cord injury

Scaffold material	Drug	Cell	Model	Results based on immune response	References
*Small molecule drugs*					
Alginate/PLGA microspheres	Minocycline and paclitaxel	–	Rat T8–T10 hemisection	Decreased ED1+ cells	171
Dextran sulfate	Minocycline hydrochloride	–	Rat C5 contusion	Reduced CD68+ cells, the percentage of M1 cells (%M1), M1/M2 ratio but no significant change of %M2	172, 173
3D‐biodegradable porous hybrid nanoscaffolds (Chitosan‐manganese dioxide)	Methylprednisolone	–	In vitro THP1 monocytes, Mouse T8 hemisection	Reduced expression of pro‐inflammatory cytokine genes (TNF, IL1b, IL6, CCL2, and CCL5) in vitro and in vivo; Reduced CD11b+ macrophage infiltration and glial scar in vivo	174
Agarose/PLGA‐nanoparticles	Methylprednisolone	–	Rat T9–T10 contusion	Reduced number of ED1+ cells is correlated with the diffused drug; Diminished the expression of pro‐inflammatory related proteins including Calpain and iNOS	175
AC/PMMA‐nanoparticles	Mimetic‐drug compounds (To‐Pro3)	–	Mouse T11 compression	Selective uptake of the PMMA‐NPs by the activated CD11b+ microglia/macrophages	176
Glycol chitosan‐oxidized HA	Tauroursodeoxycholic acid	–	Rat T9 contusion	Decreased pro‐inflammatory cytokines (TNF α, IL‐1 β, IL‐6) and GFAP	177
PLGA–PEG–PLGA	Baricitinib	–	Rat SCI	Inhibited the phosphorylation of JAK2, STAT3 and suppressed the production of inflammatory cytokines; Inhibited M1 polarization in microglia	178
RADA16‐FGL	Taxol	–	Rat T9 contusion	Reduced CD68+ and GFAP+ cells	179
Acellular spinal cord scaffold	Bisperoxovanadium	–	Rat T9–T10 hemisection	Enhanced M2 polarization and decreased M1 polarization	180
Injectable PEG‐diacerein/Graphene oxide	Diacerein	–	In vitro BV‐2 microglia and astrocytes, Rat T9 compression	Decreased the microglial LPS‐induced inflammation and astrocytes hyperactivation; Decreased astrocytic scar area in vivo	181
Hybrid Fmoc‐grafted chitosan/Fmoc‐IKVAV hydrogel	Curcumin	–	Rat T9 transection	Increased Arg1+ cells and percentage of Arg1+/CD68+	182
Electrospun PLLA	Ibuprofen and triiodothyronine (T3)	–	Rat T8 contusion	Decreased glutamate release and percentage of astrocytes	183
Glycol chitosan and oxidized hyaluronate	Gold nanoparticles conjugated with ursodeoxycholic acid	–	In vitro bone‐marrow‐derived macrophages, Rat T9 compression	Combined with NIR, increase in local temperature decreased NO, TNF α, IL‐1 β Decreased CD68, CD86, TNF‐α, IL‐1 β through MAPKs in vivo	184
*Proteins/peptides*					
Collagen	NT3	–	Rat T9–T10 hemisection	Reduced macrophage/microglial activation (Iba1, NG2)	185
HA/MC	Anti‐inflammatory peptide KAFAKLAARLYRKALARQLGVAA (KAFAK) and BDNF	–	Rat T10 compression	Reduced pro‐inflammatory cytokines expression (TNF‐α, IL‐1β, IL‐6) and glial scar formation; Increased IL‐10 expression	186
Chitosan–Collagen	Serp‐1	–	Rat T10 dorsal column crush injury	Reduced CD3+ T Cell Infiltration, no effect on F4/80 macrophages	187
Functionalized peptides: RADA16‐IKVAV/ RADA16‐RGD	Cocktail of growth factors	–	Rat T9 transection	Induced the populating CD68+/IBA1+ macrophages/microglia cells into M2 phenotypes, producing anti‐inflammatory factors.	188
PLGA–PEG–PLGA	Milk fat globule–epidermal growth factor 8	–	Rat T9–T10 crush injury	Promoted microglia conversion to M2 type; Decreased CD68+ macrophages and iNOS+ cells; Increased CD206+ cells	189
*Nucleic acids*					
Aligned PCLEEP fiber‐collagen	miR‐219/miR‐338	–	In vitro rat microglia, Rat C5 hemisection	Decreased activation of microglia and astrocytes in vitro; Decreased expressions of TNF‐α and GFAP in vivo.	190
PLG multichannel bridge	IL‐10 and IL‐4 encoding lentiviral vectors	–	Mouse T9–T10 hemisection	Induced polarization of macrophages to M2 phenotype	191
*Extracellular vesicles*					
Injectable F127–polycitrate–polyethyleneimine hydrogel	hMSC‐extracellular vesicles	–	Rat T10 transection	Reduced fibrotic scar, CD68+, and Iba1+ cells	192
PPFLMLLKGSTR‐HA	hMSC‐exosomes	–	Rat T9–T10 transection	Decreased expression of iNOS, damage 4‐hydroxynonenal (HNE) and 8‐hydroxy‐2′‐deoxyguanosine (OHdG)	193
*Combinations of small molecules/proteins/nucleic acids*					
Microenvironment‐responsive microsol electrospun fiber scaffold	IL‐4 plasmid‐loaded liposomes, NGF	–	Rat T9 hemisection	Sequential release of plasmids and NGF shifted immune cells subtype to down‐regulate the acute inflammation response, promoted the polarization of local microglia/macrophages to M2 phenotype, reduced scar tissue formation	194
Self‐assembling RGD‐PEG‐maleimide hydrogel depot	Methylprednisolone sodium succinate, bFGF, BDNF, and VEGF	–	Rat T10–T11 contusion	Reduced Iba1 and CD68 expression RNA‐Seq shows reduced expressions of macrophages, monocytes, neutrophils, T‐lymphocytes, B‐lymphocytes, microglia markers	195

*Note*: The phenotypes of macrophages and microglia are presented as reported by the respective studies. In these studies, M1 typically refers to the pro‐inflammatory phenotypes whereas M2 typically refers to the anti‐inflammatory phenotypes.

**TABLE 3:** Selected scaffolds for cell delivery with immunomodulation features after spinal cord injury

Scaffold material	Drug	Cell	Model	Results based on immune response	References
*Scaffolds with cells*					
Cell‐adaptable neurogenic hydrogel	–	ADSCs	Rat T9–T10 transection	Recruited macrophages toward M2 phenotype with M2 macrophages containing exosome and increased expression of CD206; Reduced IL‐6, pAkt, and IL‐6/PI3K/Akt signaling	199
PLGA scaffold	–	hMSCs	Rat T9–T10 hemisection	hMSCs survived well with PLGA scaffold; Diminished presence of CD3+ T‐cells; Mitigated invasion of iNOS‐carrying mononuclear leukocytes; Reduced number of CD68+ microglia/macrophage	200
PLGA scaffold	–	hMSCs	Rat T9–T10 hemisection	Soft scaffold with hMSCs reduced neural inflammatory markers of CD11b, nitrotyrosine (a marker of oxidative protein nitration), and GFAP	201
Fibrous PGA scaffold	–	hNPCs	Rat T10–T11 hemisection	Reduced microglia/macrophage infiltration; Polarized microglia/macrophage from M1 to M2 type	202
Acellular spinal cord scaffold	–	bMSC	Rat T9–T10 hemisection	Decreased numbers of CD68+ macrophages (microglia) and CD6+ T cells	203
Recombinant spider silk protein (spidroin) and HA hydrogel	–	hNPC	In vitro human peripheral blood mononuclear cells	Spidroins but not with HA hydrogel increased the proportion of activated CD69+ CD4+ T cells, CD8+ T cells, B‐cells, and NK cells	204
Agarose/Carbomer/PEG+RGD + ECM	–	hMSC	Mouse T12 compression	Increased amount of recruited macrophages; 10‐fold increase of Arginase I transcript	205
GelMa	–	miNSCs	Mouse T9–T10 transection	Reduced CD68+ reactive macrophages/microglia at the lesion site and at the rostral and caudal regions; inhibited astrocytic scar formation	206
Spinal cord‐derived ECM hydrogel	–	Stem cells from human apical papilla	In vitro mouse microglia	Increased Arg1 expression; Decreased Nos2/Arg1 ratio	207
Decellularized spinal cord/electrospun PLGA shell	–	Rat NSC	Rat T10 transection	Induced macrophage/microglia polarization toward M2 phenotype; Increased CD206+ cells and CD206/CD86 ratio	208
Nerve‐guide collagen scaffold	–	Rat MSC	Rat T9 hemisection	No infiltrated neutrophils and lymphocytes; Induced M2 polarization (reduced CD68 and iNOS, increased CD206)	209
*Scaffolds with both drugs and cells*					
Fibrin hydrogel	Lycium barbarum oligosaccharide (LBO)	Nasal mucosa‐derived MSCs (EMSCs)	Rat T10 transection	Promoted microglia M2 polarization through PI3K–Akt–mTOR pathway	210
Pluronic F‐127, heparin	bFGF	Dental pulp stem cells	Rat T9 crush injury	Decreased microglia/macrophages activation and pro‐inflammatory cytokine (IL‐6, NF‐κB, TNF‐α)	211
Agarose/Carbomer/PEG+RGD + ECM	human chemokine (C—C motif) ligand 2 (hCCL2)	hMSC	Mouse T12 compression	Increased macrophages recruitment and conversion to M2 phenotype	212

*Note*: The phenotypes of macrophages and microglia are presented as reported by the respective studies. In these studies, M1 typically refers to the pro‐inflammatory phenotypes whereas M2 typically refers to the anti‐inflammatory phenotypes.

Substrate stiffness is a major contributing factor besides materials chemistry in triggering gliosis from astrocytes and microglia around implants in the CNS. A stiff substrate with a modulus of 30 kPa could activate both astrocytes and microglia into pro‐inflammatory phenotypes and secreted more TLR4, PPARγ, Caspase‐1, and IL‐1β, as compared to the more compliant substrate (100 Pa).^251^, ^252^ Likewise, increased astrogliosis and upregulation of inflammatory proteins were found in astrocytes on stiff substrates with moduli of 8 or 30 kPa as compared to the compliant 100–200 Pa soft substates.^251^, ^253^ Interestingly, A1 type reactive astrocytes with increased expression of IL‐1β and GFAP were observed in 3D soft hydrogel (43 Pa as compared to 991 Pa) instead,^254^ suggesting the differences in modulus range and model dimension could lead to contrasting astrocyte response toward substrate stiffness. As the glial scar is also softer than the healthy spinal cord tissue and is correlated with astrocyte reactivity,^255^ it is important for the scaffold to have a stiffness that matches the native tissue. In addition, regenerative approaches that involve glia scar digestion should also be cautious of the effect of matrix softening on astrocyte activation.

In general, softer or physiologically compliant scaffolds appear to induce less immune cells activation and pro‐inflammatory cytokines secretion. The future scaffold design could also explore manipulating the invading peripheral immune cells through scaffold stiffness.

#### 4.2.5 Porosity and surface topography

Apart from having a tissue‐compliant stiffness, for better integration with host tissue and to provide contact guidance, scaffolds are usually designed to allow efficient cell infiltration, in which pore size was also found to regulate macrophage phenotypes.^256^, ^257^, ^258^, ^259^ Otherwise, the scaffolds may elicit FBR, which in turn leads to larger glial scar or cyst formation. In addition to the macroarchitecture of the scaffolds, the microarchitecture of the scaffolds is also crucial in modulating the immune response through the surface topography of the implants.^231^ The responses of neural cells toward surface topography are frequently exploited for neural tissue engineering but less consideration has been placed on the inflammatory response post‐SCI.^260^

Macrophage phenotype can be modulated by regulating cell shape through micro or nanopattern topographical cues.^261^ Specifically, the elongated macrophages on the 400–500 nm wide nanopatterned grooves were driven toward an anti‐inflammatory phenotype.^262^ Similarly, electrospun nanofiber scaffold has served as an alternative to providing topographical stimuli. In particular, a reduced number of macrophages, macrophage activation, and secretion of pro‐inflammatory molecules were found on PLA nanofiber (ø 600 nm) scaffolds as compared to films and microfibrous (ø 1.6 μm) scaffolds.^263^ Similar results were also observed with PCL scaffolds. As compared to PCL films and random nanofibers, the aligned nanofibers (ø 506 nm) scaffolds had reduced monocyte/macrophage adhesion and a thinner fibrous capsule in vivo.^264^ Recently, in a transplanted nanofiber‐hydrogel composite scaffold for SCI treatment, anti‐inflammatory macrophages were found to be present predominantly in the areas with the nanofibers, suggesting the possible role of nanofibers directly modulating immune cells phenotype.^228^ On the other hand, while less is known about regulating lymphocytes and neutrophils through surface topography, lymphocytes and neutrophils found on implants with rough surfaces, created through sandblasting followed by acid‐etching or physical scratching, secreted less pro‐inflammatory cytokines.^265^, ^266^, ^267^ In particular, rough and hydrophilic surfaces polarized the adaptive immune system toward the pro‐regenerative Th2 phenotype mediated by macrophages.^267^

Similar to macrophages, nanofiber topography has a positive effect on astrocytes as nanofiber topography promoted astrocyte adhesion with downregulated GFAP expression, leading to reduced astrocytes activity.^239^ Aligned electrospun fiber topography (ø 2.4 μm) also directed astrocytic migration and increased the rates of glutamate uptake as a readout for neuroprotective effect.^268^ Conversely, aligned PLA microfibers (ø 1.8 μm) mildly induced cytotoxic A1 phenotype, which could be alleviated by the presence of transforming growth factor β3 (TGFβ3).^269^ For microglia, a higher concentration of the pro‐inflammatory cytokine TNF‐α was detected in culture media on fibers (ø 1.1 μm) than on films.^270^ This suggests that while microglia and macrophage are performing similar functions in phagocytosis, the response of these cells to the surface topography is different.

## 5 FUTURE PERSPECTIVES AND CONCLUSIONS

SCI elicits an inflammatory cascade that exerts a complex and dynamic microenvironment within the spinal cord tissue. Although substantial advances have been made to identify the cellular and molecular pathways that shape the immunological responses after SCI, appropriate interventions that involve the use of stem cells and/or biomaterials are necessary to avoid enhanced neuroinflammatory events that may derail tissue regeneration and recovery. While there remain limitations and challenges to current SCI therapies including the route of drug delivery to alleviate the immune responses, there are currently alternative approaches that increase the permeability into the BSCB through microbubble‐assisted focused ultrasound.^271^ However, evaluation of the safety of such a strategy in human is underway, and clinical usage would require precise control over parameters to reduce inflammatory responses, glial cell activation, and tissue damage.^272^

**FIGURE 2:**
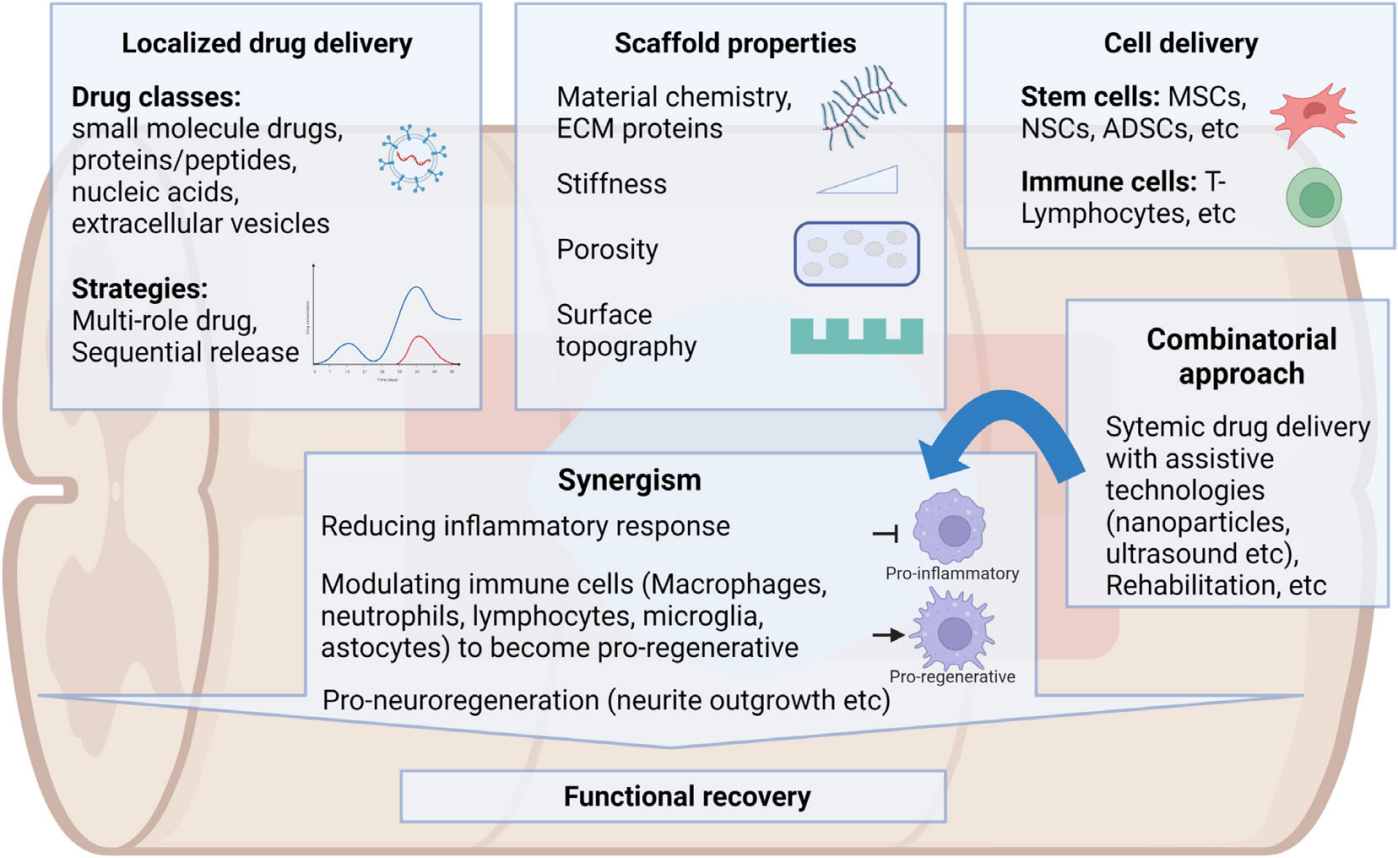
Biomaterial‐based therapies to modulate neuroinflammation and treat SCI. The combination of biomaterial design, drug delivery, cell therapy, and rehabilitation can be utilized to target neuroinflammation and neuroregeneration to achieve a synergistic effect in promoting functional recovery after SCI. Illustrations are created with BioRender.com.

On the other hand, the future scaffold for treating SCI should include immunomodulation design to work synergistically with the strategies that promote nerve regeneration through neurite outgrowth, remyelination, and reduced glial scarring (Figure 2). Physical and chemical characteristics of the material for better immunomodulation outcomes should be included in future scaffold designs. Specifically, the combination of material chemistry (biocompatible), scaffold macroarchitecture (porous), surface topography (nanofibrous), surface coating (with favorable cell adhesion sites), stiffness (tissue stiffness‐matching), will likely give a favorable control for the immune response.^231^, ^273^, ^274^ We will also expect to see more systemic anti‐inflammation or immunomodulation drug administration to synergistically enhance nerve regeneration with existing neural tissue engineering therapies.^275^, ^276^ Furthermore, other newer immunomodulation drugs (parthenolide,^277^ 14‐3‐3t,^278^ miR‐194^279^) and cell transplantations (olfactory ensheathing cells,^280^ T‐lymphocytes^281^, ^282^) can be further explored and incorporated in the future strategies. In particular, thiazolidinediones and miR‐124^283^, ^284^, ^285^, ^286^, ^287^ have demonstrated the ability to target both inflammatory response and neuronal differentiation making them promising candidates to be combined with scaffold‐mediated delivery approaches for treating SCI. Since the inflammation and regeneration processes involve different stages and different cell populations, scaffolds with a sequential delivery mechanism of drugs or physical signals targeting different stages could be more effective in promoting nerve regeneration and motor recovery after SCI.^194^, ^288^

Current immunomodulation approaches for treating SCI are mainly through immune response reduction and macrophage phenotypic shift.^289^, ^290^, ^291^, ^292^ It will be valuable to assess other immune cells and responses as well as target these mediators for better nerve regeneration. As discussed earlier, future scaffold designs may benefit from referring to the biomaterial approaches in targeting autoimmune diseases, graft rejection, and inflammation in other tissues.^216^, ^282^, ^293^, ^294^, ^295^, ^296^, ^297^

Finally, including a rehabilitation regimen would also be beneficial as rehabilitation and scaffold implantation was found to synergistically promote the skewing of macrophage phenotype toward anti‐inflammatory phenotypes and better functional recovery.^298^, ^299^ A combinatorial approach will increase the likelihood of more successful immunomodulation and consequently functional recovery after SCI.

## AUTHOR CONTRIBUTIONS

**Cheryl Lee:** Conceptualization (equal); investigation (equal); writing – original draft (equal); writing – review and editing (equal). **Wai Hon Chooi:** Conceptualization (equal); investigation (equal); writing – original draft (equal); writing – review and editing (equal). **Shi Yan Ng:** Conceptualization (equal); supervision (equal); writing – review and editing (equal). **Sing Yan Chew:** Conceptualization (equal); funding acquisition (equal); supervision (equal); writing – review and editing (equal). All authors approved this manuscript for publication.

## CONFLICT OF INTEREST

The authors declare no conflict of interest.

### PEER REVIEW

The peer review history for this article is available at https://publons.com/publon/10.1002/btm2.10389.

## Data Availability

Data sharing is not applicable to this article as no datasets were generated or analyzed during the current study.
